# Evolution and Engineering of Precisely Controlled Ge Nanostructures on Scalable Array of Ordered Si Nano-pillars

**DOI:** 10.1038/srep28872

**Published:** 2016-06-29

**Authors:** Shuguang Wang, Tong Zhou, Dehui Li, Zhenyang Zhong

**Affiliations:** 1 State Key Laboratory of Surface Physics and Department of Physics, Collaborative Innovation Center of Advanced Microstructures, Fudan University, Shanghai 200433, China; 2 School of Science, Shandong University of Technology, Zibo 255049, China; 3 Shanghai Institute of Applied Physics, Chinese Academy of Sciences, Shanghai 201800, China

## Abstract

The scalable array of ordered nano-pillars with precisely controllable quantum nanostructures (QNs) are ideal candidates for the exploration of the fundamental features of cavity quantum electrodynamics. It also has a great potential in the applications of innovative nano-optoelectronic devices for the future quantum communication and integrated photon circuits. Here, we present a synthesis of such hybrid system in combination of the nanosphere lithography and the self-assembly during heteroepitaxy. The precise positioning and controllable evolution of self-assembled Ge QNs, including quantum dot necklace(QDN), QD molecule(QDM) and quantum ring(QR), on Si nano-pillars are readily achieved. Considering the strain relaxation and the non-uniform Ge growth due to the thickness-dependent and anisotropic surface diffusion of adatoms on the pillars, the comprehensive scenario of the Ge growth on Si pillars is discovered. It clarifies the inherent mechanism underlying the controllable growth of the QNs on the pillar. Moreover, it inspires a deliberate two-step growth procedure to engineer the controllable QNs on the pillar. Our results pave a promising avenue to the achievement of desired nano-pillar-QNs system that facilitates the strong light-matter interaction due to both spectra and spatial coupling between the QNs and the cavity modes of a single pillar and the periodic pillars.

The semiconductor system of nano-cavity embedded with semiconductor QNs as the active media has attracted tremendous attention^1^. It offers an ideal platform to study fundamental properties associated with the cavity quantum electrodynamics^2^,^3^. Particularly, thanks to the efficient heat sinking and straightforward carrier injection^4^,^5^, high extraction efficiency and clean Gaussian far-field emission^6^,^7^, pillar-QNs semiconductor system exhibits great potential in innovative devices. They have been implemented in some unique optoelectronic devices, such as single photon source^6^,^7^,^8^ and laser diode^5^, which are key components for quantum information processing^9^, quantum communication^10^,^11^ and integrated photon circuits^12^,^13^. Another advantage of the pillar-QNs system is the versatile modifications on them. On one hand, the pillar is readily tailored in aspects of the size, the shape, and the arrangement to achieve light management within the pillar, high extraction efficiency and directional output of light without using waveguide^4^,^6^,^7^. Two coupled nano-pillars in the form of a ‘photonic molecule’ were also designed to produce ultra-bright entangled photon pairs^14^. On the other hand, the electronic states of QNs embedded in the pillar can be feasibly manipulated through quantum confinement effect^15^, which is intimately associated with their size, shape, composition and etc. They can also be tuned by the quantum confinement Stark effect^16^. Thus, the spectra coupling between the QNs and the cavity mode of pillars can be readily achieved. Particularly, the controlled coherent coupling of three spatially separated QDs via the cavity mode in micro-pillar has also been observed^17^. To ultimately realize the strong light-mater interaction, the spatial coupling between the QNs and the cavity mode of pillars is required as well^2^. In general, the QNs should be precisely positioned at the desired site in the pillar, where the cavity modes of the pillar mainly locate. The site-controlled QD in the pillar can be obtained via post-growth etching processes^8^, which is complex particularly for the precise site-controlling. The site-controlled QD-in-nanowires have also been realized by selective growth^18^,^19^. Whereas, such QDs always locate at the center of the pillar or the nanowire. It is generally not optimum for the strong light-matter interaction since most cavity modes, *e.g.* whispering gallery modes (WGMs)^5^, helically propagating cavity modes (HPCMs)^20^, and Mie resonance modes^21^, mainly locate near the periphery of the pillar. Moreover, the array of nano-pillars embedded with QDs have also attracted much attention for the prospect in understanding the coupling between nano-pillars^14^, exploiting the effects of two-dimensional photonic crystal^22^ and the realization of the arrays of integrated quantum light sources^6^,^12^. Accordingly, to fully explore and utilize the light-matter interaction in the hybrid pillar-QD system, array of nano-pillars embedded with QDs near the pillar periphery is highly desirable. However, the general issues of how and why the QNs can be precisely controlled on the pillar have not been comprehensively addressed. A scalable array of desired nano-pillars in a large area is also a big challenge.

In this report, we present a feasible route to fabricate ordered Si nano-pillars with controlled diameter and periodicity in a large area. By deliberately adjusting the growth conditions, three typical Ge QNs regimented on the top edge of the Si nano-pillars can be intentionally realized. Such hybrid pillar-QN system is the potential candidates for the innovative optoelectronic devices due to the compatibility with sophisticated Si integration technology^23^. The comprehensive scenario of the non-uniform growth of Ge on the Si pillar is disclosed in terms of the strain relaxation and the thickness-dependent anisotropy of surface diffusion of Ge adatoms, which is intimately associated with the surface chemical potential (SCP) on the pillar. It provides the key insight into the controlled self-assembly of various QNs on the pillar. Furthermore, it inspires a two-step growth procedure to efficiently tailor the self-assembled nanostructures on the pillar in a deliberate way. Our results offer a promising and feasible route to the desired nano-pillar-QNs semiconductor systems that are persistently pursued for the fundamental studies of the cavity quantum electrodynamics and the fabrication of the innovative nano-optoelectronic devices.

## Results

**Figure 1:**
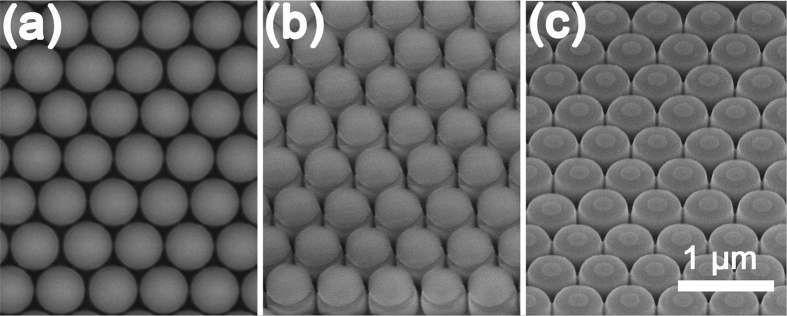
Typical SEM images during the fabrication of ordered Si nano-pillars via nanosphere lithography, (**a**) a template of self-assembled monolayer of PS nanosphere on a clean Si substrate, (**b**) ordered Si pillars with remained PS nanosphere on top after dry-etching of Si, (**c**) ordered Si pillars after removal of the rest nanosphere mask.

The scalable array of ordered Si nano-pillars in a large area is obtained via Nanosphere lithography. Figure 1a is the typical scanning electron microscope (SEM) image of a monolayer of polystyrene (PS) spheres compactly arranged on a Si (001) substrate, which is obtained via the modified Langmuir-Blodgett technique^24^. The PS spheres are well ordered in a hexagonal lattice. Figure 1b shows the typical SEM image of ordered Si pillars with a PS sphere on each pillar after reactive ion etching (RIE) of Si. It indicates that the PS sphere is a good mask to obtain Si pillars. Figure 1c shows the typical SEM image of ordered Si pillar after the final removing of PS sphere by the oxygen plasma. The period of Si pillars is the same as the diameter of the PS sphere. Thus, it is readily controlled by using the PS spheres of a desired diameter in the range of 100 nm to several micrometers. The height and the diameter of the Si pillars can be intentionally adjusted by tuning the RIE conditions. Moreover, the area of ordered Si pillars is scalable up to square centimeters except for some domain boundaries.

**Figure 2:**
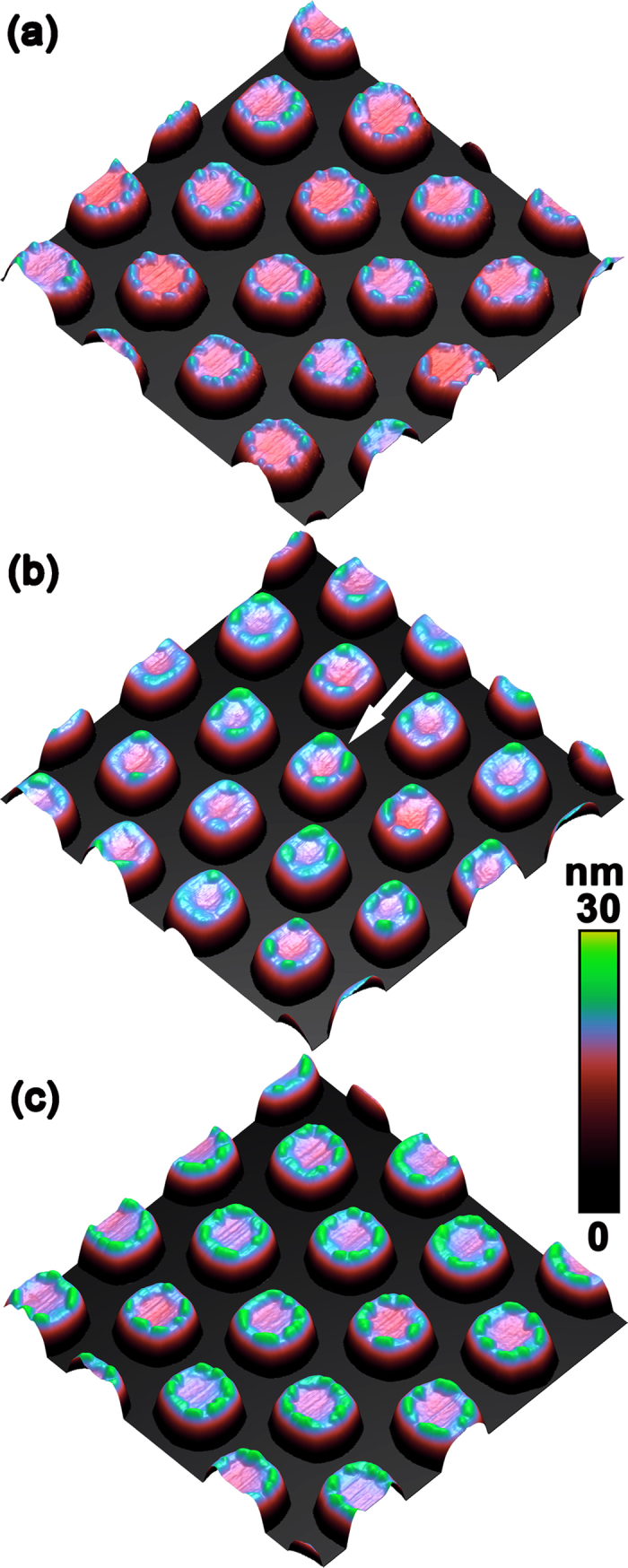
AFM images (2 × 2 μm^2^) of self-assembled Ge nanostructures on the ordered Si nano-pillars via the one-step procedure at 520 °C and a Ge growth rate of 0.025 Å/s with nominal Ge deposition of, (**a**) 1.8 nm, (**b**) 2.0 nm, (**c**) 2.2 nm. The arrow in (**b**) denotes a molecule of four QDs on the pillar. The color bar is shown at the right.

Figure 2 show the atomic force microscopy (AFM) images (2 × 2 μm^2^) of the surface morphologies after 1.8, 2.0 and 2.2 nm Ge deposition on the pillar of 310 nm in diameter and 490 nm in period. All Ge is grown via a one-step procedure with the effective growth rate of 0.025 Å/s at 520 °C. For 1.8 nm Ge deposition, circularly arranged small QDs appear at the top edge of each pillar, which is like a QDN, as shown in Fig. 2a. This result is consistent with the previous one^25^. Whereas, the number and the size of QDs in the present case are smaller, which are mainly due to the smaller diameter of pillar and the low growth temperature. It can be found that each QDN contains ~9 QDs. All QDs are hut-clusters^26^ of 4.2 ± 1.1 nm in height. For 2.0 nm Ge deposition, large QDs of 6.3 ± 1.2 nm in height appear on the pillars with the decreased QD number of ~3. On some pillars, four QDs appear, as denoted by an arrow in Fig. 2b. Such arranged QDs can be regarded as a QDM^25^,^27^. The increase of the QD size and the decrease of the QD number are attributed to the coarsening by the Ostward ripening and/or the coalescence of QDs during further Ge deposition^28^. For 2.2 nm Ge deposition, the Ge QDs at the top edge of the pillar are still distinguishable, as shown in Fig. 2c. The number and the height of QDs on the pillars are ~6 and 6.8 ± 0.7 nm, respectively. Evidently, more QDs are formed again rather than the pronounced further increase of QD size. This result is mainly due to the self-limiting growth of QDs^29^. In addition, the QDs tend to connect together at their base, where ring-like structure begins to appear. These results demonstrate that the amount of deposited Ge considerably affects the configuration of the self-assembled Ge QDs on the Si nano-pillars. On the reference flat Si (001) substrates, pyramid-like and dome-like QDs are obtained after 1.8, 2.0 and 2.2 nm Ge deposition under the same growth conditions (see Supplementary Fig. S1a–c)^28^,^29^,^30^. All QDs are spatially random. Due to sufficient Ge deposition, most of QDs are dome-like, and even some superdome appears^30^.

**Figure 3:**
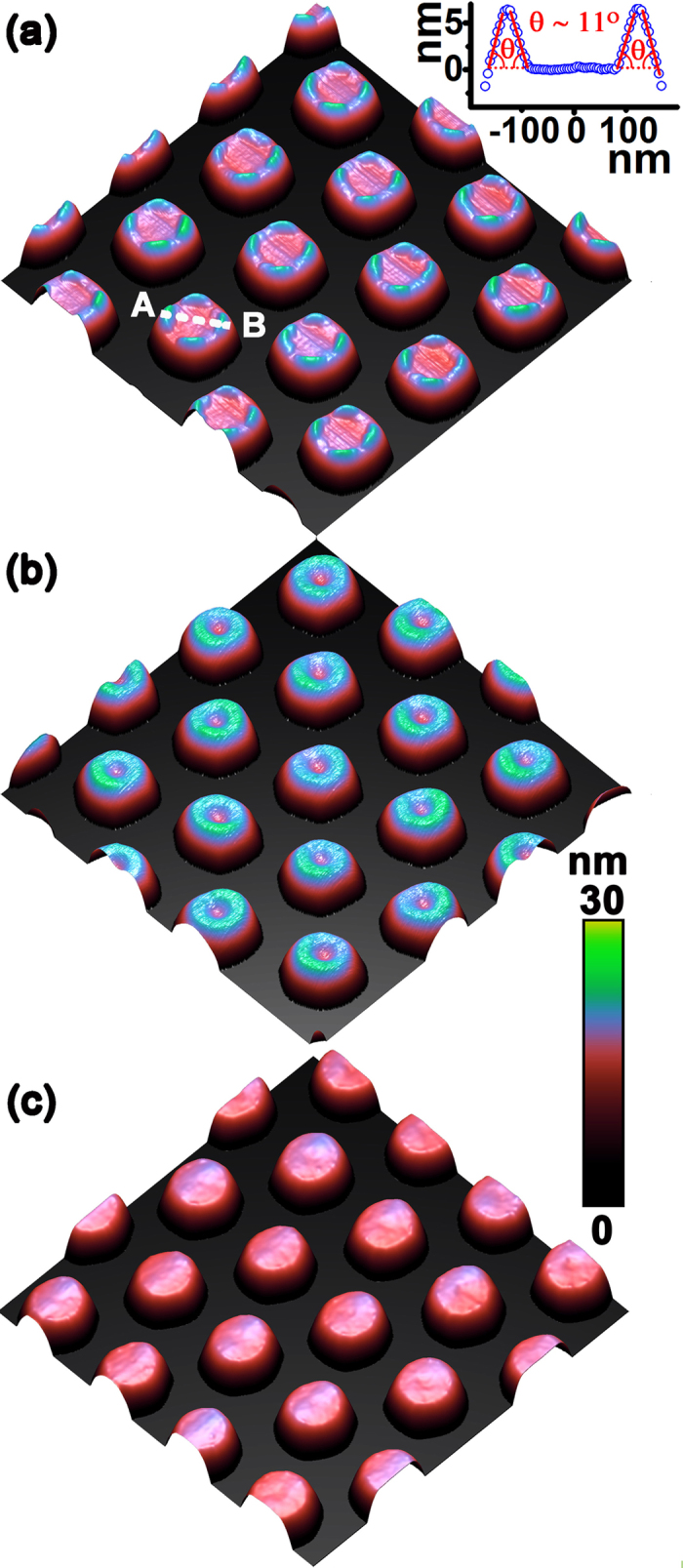
AFM images (2 × 2 μm^2^) of self-assembled Ge nanostructures on the ordered Si nano-pillars via the two-step procedure with the Ge deposition of, (**a**) (0.8 nm at 500 °C and 0.1 Å/s) + (1.0 nm at 520 °C and 0.025 Å/s), (**b**) (0.8 nm at 500 °C and 0.1 Å/s) + (1.0 nm at 580 °C and 0.025 Å/s), (**c**) (0.8 nm at 580 °C and 0.025 Å/s) + (1.0 nm at 480 °C and 0.1 Å/s). The inset in (**a**) shows the height profile along the dotted line AB in (**a**). The color bar is shown at the right.

**Figure 4:**
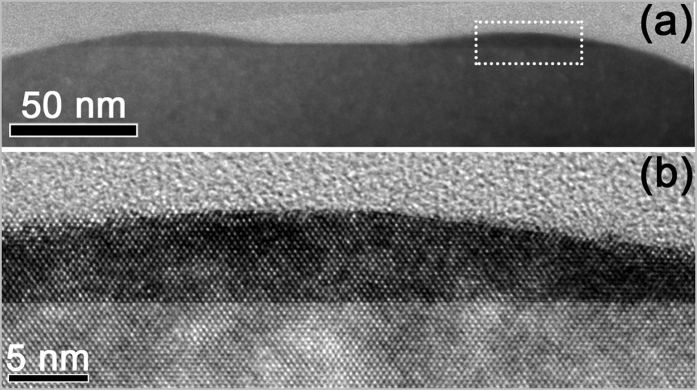
(**a**) XTEM image of two QDs of a QDM on a Si nanopillar, (**b**) high-resolution XTEM image of the region denoted by the dashed box in (**a**).

It is well known that the growth conditions remarkably affect the self-assembled QDs on the flat substrates^31^. To deliberately tailor the self-assembled nanostructures on the nano-pillar, we develop a two-step procedure for the Ge growth. Figure 3a shows the AFM image (2 × 2 μm^2^) of the surface morphology after 1.8 nm Ge deposition on the pillar via the two-step growth procedure. Firstly, 0.8 nm Ge is grown at 500 °C with a growth rate of 0.1 Å/s. In the second step, another 1 nm Ge is grown at 520 °C with a growth rate of 0.025 Å/s. The growth interruption between these two steps is about 1 minute. Obviously, at the top edge of each pillar, four uniformed QDs appear and form a QDM. They exhibit four-fold symmetry along <100> direction. The QDs are hut-clusters of 6.3 ± 0.3 nm in height. The height profile in the inset of Fig. 3a clearly exhibits the intersection angle of ~11° between the sidewalls of QDs and the (001) surface, which demonstrates the {1 0 5} facets of the QDs. The cross-sectional transmission electron micrograph (XTEM) image of two QDs of a QDM on a Si nanopillar is shown in Fig. 4a. The high resolution XTEM image in Fig. 4b demonstrates that the Ge QD is crystalline and defect-free. On the reference flat Si (001) substrate, dome-like QDs are randomly distributed (see Supplementary Fig. S2a). Using the similar two-step growth procedure except for the higher growth temperature of 580 °C in the second step, we readily obtain a QR on each pillar, as shown in Fig. 3b. The cross-section of the QR is a triangle with a height of 5.6 ± 0.5 nm and a half-width of 44.0 ± 1.7nm. On the reference flat Si (001) substrate, large dome-like QDs are randomly distributed (see Supplementary Fig. S2b). In the contrary case, the first 0.8 nm Ge is grown at 580 °C with a growth rate of 0.025 Å/s, then in the second step another 1 nm Ge is grown at 480 °C with a growth rate of 0.1 Å/s. Surprisingly, no QD appears on each pillar, although dense hut-clusters and some domes are observed on the reference flat Si (001) substrate (see Supplementary Fig. S2c).

Our results manifest that the QDN, the QDM and the QR can be realized on the nano-pillars under the proper growth conditions. In addition, it is disclosed that the QDN tends to evolve to the QDM and finally to the QR with the increase of the deposited Ge, as shown in Fig. 2a–c. Such nanostructures and their evolutions with the Ge deposition are distinct from that of QDs on the flat substrates^30^,^31^. This is mainly attributed to the preferential site of QNs at the top edge of the pillar and the partial strain relaxation there. More interestingly, with the same nominal Ge deposition, the QDN, the QDM, the QR and even no QNs can be intentionally realized on the same pillar by adjusting the growth conditions, as shown in Figs 2a and 3a–c, respectively. Such different configurations of QNs with the same nominal Ge deposition on the pillars indicate that the actual amount of Ge at the top edge of pillar is different.

## Discussion

**Figure 5:**
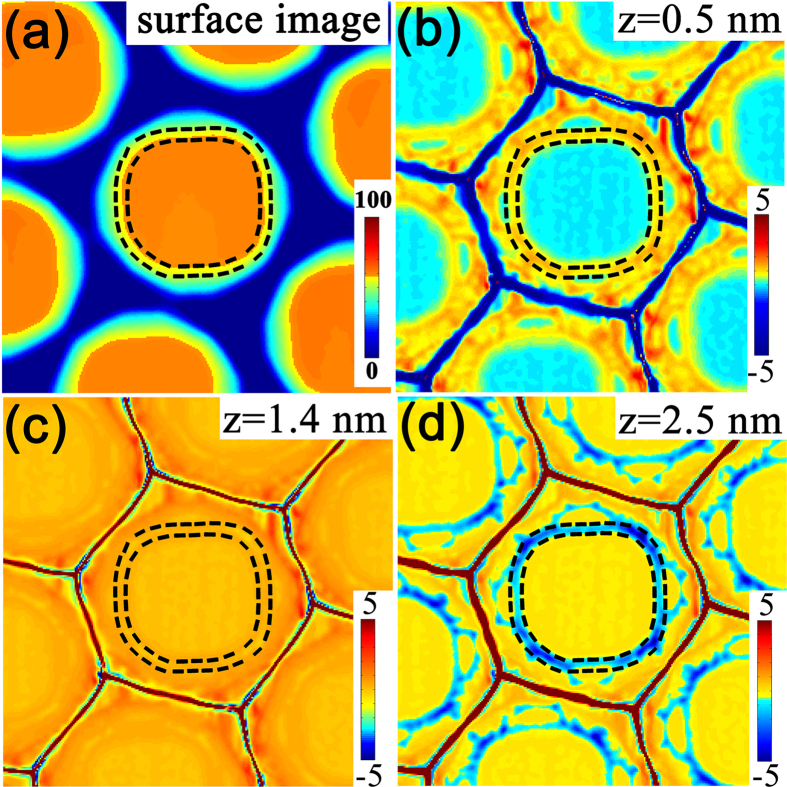
(**a**) Typical AFM images (1 × 1 μm^2^) of the ordered Si nano-pillars before Ge growth, (**b**–**d**) the corresponding SCP around the pillars with the parameter Z of 0.5 nm, 1.4 nm, and 2.5 nm, respectively. The two black dashed circles denote the top edge of the pillar.

Bulk-like Ge thick layer has been obtained on the very large Si pillar with the size of several micrometers at a very high growth rate and a relatively low growth temperature^32^. They are completely distinct from the obtained nanostructures and the related growth conditions in our cases. To disclose the inherent mechanism underlying those unique features, we systematically analyze the actual distribution of deposited Ge around the pillar based on the SCP and its variation with the Ge deposition. The SCP is estimated by^33^,^34^,


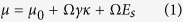



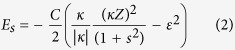


where *μ*_*0*_ is the SCP of the flat surface, *Ω* is the atomic volume, *γ* is the surface energy per unit area, *κ* is the surface curvature, *E*_*s*_ is the local strain energy density, *C* is the elastic constant, Z is the parameter corresponding to the nominal thickness of the Ge film and depending on the actual surface undulation, *ε* is the misfit strain. The factor (1 + s^2^)^−1/2^ denotes the dependence of the nominal thickness on the slope (*s*) due to the different growth rate around the pillar. During the MBE growth, the Ge film all over the pillar is not uniform without the surface diffusion of adatoms. Given the nearly normal incidence of the molecule beam, the actual nominal thickness of Ge film around the pillar can be described as Z(1 + s^2^)^−1/2^, where the local slope s is defined to be s = tan(β). The β is the inclination angle of the local surface on the pillar with respect to the top surface of the pillar, which can be derived from the AFM data. The second and the third term in Eq. 1 represents the surface curvature contribution and the strain contribution to the chemical potential, respectively. *E*_*s*_ is the local strain-relaxation energy with respect to a flat film. Based on the height profiles, h(x, y), extracted from the AFM data, the sectional surface curvature ***κ***can be obtained by^33^,^34^


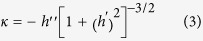


where 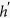 and 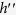
are the first and second derivatives of *h* with respect to the positions. For a given two-dimensional point *h*(x, y), the surface curvature ***κ*** (*x*, *y*) is the average of the two principal sectional curvatures obtained from Eq. (3) 34. The surface morphologies around pillars before Ge growth are shown in Fig. 5a. For simplicity, it is employed in all SCP calculation. In addition, *μ*_*0*_ is adopted to be 0 since the relative distribution of the SCP around the pillar is considered. The atomic volumes *Ω*, the surface energy *γ* and the elastic constant *C* of Ge is 13.6 cm^3^ mol^−1^, 1.835 J·m^−2^ and 1.03 × 10^11^ N·m^−2^, respectively^34^. The misfit strain *ε* is 0.04. The SCPs around the pillars with respect to the parameter Z of 0.5, 1.4 and 2.5 nm are shown in Fig. 4b–d. It is found that the SCP intimately depends on the thickness of Ge layer on the pillar. At the beginning of Ge growth, e.g. for Z = 0.5 nm, the SCP is dominated by the surface energy around the pillar. A local maximum of the SCP appears at the top edge of the pillar, as denoted by two black dashed circles in Fig. 5b. With the increase of Ge layer thickness, the strain energy becomes more and more important. For the proper thickness, e.g. around Z = 1.4 nm, the contribution of the surface energy to the SCP is essentially balanced by that of the strain energy. Accordingly, the SCP around the pillar top is nearly the same, as shown in Fig. 5c. Further Ge growth, *e.g.* for Z = 2.5 nm, gives rise to more pronounced contribution of strain energy to the SCP than that of the surface energy. As a result, a local minimum of the SCP appears at the top edge of the pillar, as shown in Fig. 5d.

**Figure 6:**
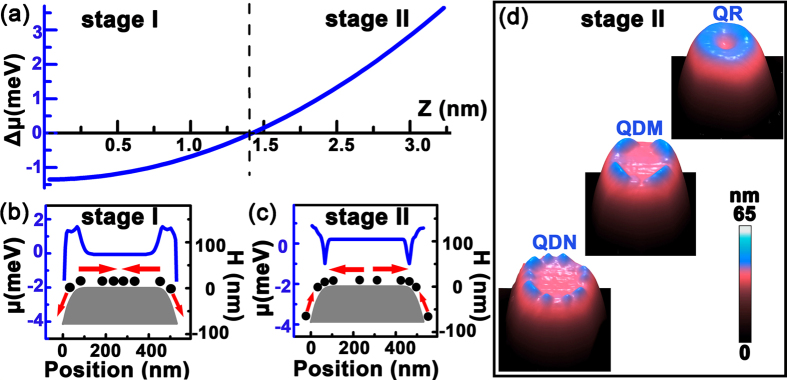
(**a**) The difference (Δ*μ* = *μ*_*center*_ − *μ*_*edge*_) of the SCP at the top center and edge of the pillar as a function of the parameter Z, (**b**,**c**) the typical SCP (blue line) and the height profile (gray region) across the center of a pillar in the growth stage I and II, respectively, (**d**) the AFM images of three typical QNs, the QDN, the QDM and the QR, on the pillar. In (**b**,**c**), the black solid circles denote the Ge adatoms, the red arrows denote the preferential diffusion of the Ge adatoms. The stage I and II denote the growth processes of Ge on the Si pillar.

Our results clearly demonstrate that the SCP at the top edge of the pillar converts from the local maximum to minimum with the Ge growth. Such conversion can be clearly represented by the difference (Δ*μ* = *μ*_*center*_ − *μ*_*edge*_) of the SCP at the top center (*μ*_*center*_) and edge (*μ*_*edge*_) of the pillar as a function of the parameter Z, which is shown in Fig. 6a. The adatoms prefer to diffuse away from the region of the local maximum SCP and incorporate in the region of the local minimum SCP. Given these unique features, the Ge growth on the pillar is distinct from the nearly isotropic growth on the normal flat substrates. In fact, it can be separated into two growth stages corresponding to the unique distribution of the SCP around the pillar top with the Ge growth, as denoted in Fig. 6a. In the stage I, *i.e.* at the beginning of Ge growth, the SCP at the top edge of pillar has a local maximum (Δ*μ* < 0). Accordingly, the Ge adatoms preferentially diffuse away from the top edge of the pillar, as schematically shown in Fig. 6b. The Ge layer at the top edge can be thinner than that around the center of pillar in this stage. In the stage II, the overall Ge layer on the pillar becomes thicker with the Ge deposition so that the SCP at the top edge of the pillar converts into a local minimum (Δ*μ* > 0). In contrast to the case in the stage I, the top edge of the pillar becomes the sink of the Ge adatoms, which can diffuse from the top center or the sidewalls of the pillar, as schematically shown in Fig. 6c. Considering the rather small region of the top edge of the pillar, as shown in Fig. 5a, the local growth rate there is much faster than the rest of the pillar. Thus, the Ge layer at the top edge of the pillar can become thicker than the rest. Accordingly, QDs can readily form at the top edge of the pillar since sufficient Ge adatoms aggregate and incorporate there to feasibly exceed the critical value. The initially formed QDs are small and preferentially locate at the top edge of the pillar, resulting in the QDN on the pillar. With the further increase of the amount of Ge at the top edge of the pillar, the QDN can evolve to the QDM due to the coarsening process^28^, and even subsequently into the QR by the connection of the neighboring QDs. The three typical QNs at the top edge of the pillar are shown in Fig. 6d.

Such comprehensive scenario of the Ge growth on the Si pillar interprets the evolution of the self-assembled nanostructures on the pillar with the amount of nominal Ge deposition, as shown in Fig. 2. It also provides the key insight into the formation of the QDM and the QR via the two-step procedure rather than the QDN via the one-step procedure with the same nominal Ge deposition. Due to the lower growth temperature and the higher growth rate in the first step of the two-step procedure, the Ge diffusion is substantially suppressed. Optimally, this first step should cover the stage I of the Ge growth on the pillar. This means that less Ge will diffuse away from the top edge of the pillar and therefore more deposited Ge can remain there in the first step. Accordingly, the QDM rather than the QDN is readily obtained on the pillar since more Ge incorporates at the top edge of the pillar via the two-step procedure. Further increase of the Ge amount at the top edge of the pillar via two-step procedure can be realized by the higher growth temperature in the second step, which mainly covers the stage II of the Ge growth on the pillar. During this stage, the enhanced surface diffusion of Ge adatoms due to the high growth temperature facilitates Ge diffusion from the top center and the sidewalls to the top edge of the pillar, where is the sink of the Ge adatoms due to the local minimum of the SCP. Consequently, much more Ge can aggregate and incorporate at the top edge of the pillar via two-step procedure, resulting in the QR there. These results indicate that the two-step procedure can considerably increase the amount of Ge at the top edge of the pillar, analogue to the increase of the total amount of deposited Ge. More interestingly, the obtained QNs via two-step procedure are much more uniform than those via one-step procedure. This may be attributed to the coincidence nucleation and growth of the self-assembled nanostructures at the top edge of the pillar, given the nearly conformal growth of Ge layer in the first step and the efficient aggregation of Ge at the top edge of the pillar in the second step. The formation of the QDM with four-fold symmetric QD in Fig. 3a is associated with the interaction between QDs and the anisotropic elastic properties^27^. Close inspection on the SCP around the pillar in Fig. 5d reveals that the SCP at the top edge of the pillar is also not uniform due to the formation of small {113} facets, which also facilitate the formation of the QDM^25^. The formation of the QR around the top edge of the Si pillar may arise from the efficient strain relaxation of the large volume of Ge there at a high growth temperature. In the contrary case, the high growth temperature and the low growth rate are adopted in the first step, while the low growth temperature and the high growth rate are adopted in the second step. This means that much Ge will diffuse away from the top edge of the pillar in the first step, and less Ge will aggregate at the top edge of the pillar in the second step due to the growth kinetic limitation^30^. As a result, much less Ge incorporates at the top edge of the pillar. For the total Ge deposition of 1.8 nm, the Ge layer all over the pillar is still below the critical thickness for the onset of the QD formation due to the lateral strain relaxation on the pillar^35^. No QD can be observed on the pillar, as shown in Fig. 3c.

Our results reveal that the self-assembled QNs at the top edge of the pillar sensitively depends on the actual Ge amount there. More importantly, the non-uniform distribution of Ge around the Si pillar top can be intentionally modulated by the tow–step procedure. Accordingly, the QDN, the QDM and the QR are readily obtained at the periphery of the Si nano-pillar. It is well known that the miniaturization of optical cavities reduces the cavity modes and increases the photon density of states, which therefore enhance light–matter interactions^5^,^36^. Given the comprehensive scenario of the Ge growth around the Si pillar top as discussed above, desired nanostructures with precise controllability are expected to be obtained even on smaller Si pillar by deliberately adjusting growth conditions. One point should be mentioned. In the extreme case that the size of QNs approaches the top diameter of the pillar, the QNs tend to coarsen to be a single QD at the top of pillar due to the efficient strain relaxation^25^. Further growth will embed this single QD at the center of pillar. This frequently occurs for the heteroepitaxial growth on the rather small nanowires^18^,^19^. Accordingly, critical growth conditions is required to realize QNs at the periphery of the small pillar or nanowire. Such QNs on the pillar can be naturally capped or embedded during the subsequent growth of pillar materials^18^,^19^. Considering the improved quantum efficiency due to the partial relaxation of the carrier transition selection rules, compatibility with the sophisticated Si integration technology and the suitable wavelength for the telecommunication and the on-chip optical interconnects, the Ge QNs has been extensively studied for the optoelectronic applications^37^,^38^. Recent studies demonstrate that the nano-pillars on a dielectric interface even with low index contrast can provide unique HPCMs^20^, which is similar to the WGMs. The Si nano-pillar on a Si-on-insulator (SOI) or a GeSi buffer layer are also expected to have the HPCM. Accordingly, the desired Si pillar (optical cavity) embedded with controllable Ge QNs (active media) may have great potential in the investigation of the fundamental features of the cavity quantum electrodynamics and the dense integration of complex nano-photonics circuit. In addition, the controllable QNs on the pillar are nearly independent on the pillar height. The precisely controlled Ge QDM and QRs are readily achieved on the short Si pillars, which can be feasibly embedded in the Si matrix. Given the good size uniformity and the precise controllability on the size, site and arrangement, the QDM on the short pillar can be a promising building block for a QD cellular automata^39^, and the QR is an ideal platform for the exploration of the magneto-optical excitations and the spintronic devices on the basis of the Rashba spin-orbit interaction and Aharonov-Bohm (AB) effect^40^. It is also noteworthy that the growth scenario of the self-assembled nanostructures on the pillar discussed above should be adaptable for other material systems, *e.g.* compound semiconductors, since the distribution and the conversion of the SCP around the pillar during the heteroepitaxy is universal.

In conclusion, ordered Si nano-pillars with controllable diameter and periodicity can be readily obtained in a large area via nanosphere lithography. By tailoring the growth conditions, self-assembled Ge QDN, QDM and QR can be intentionally realized at the periphery of the Si nano-pillars. The evolution of those nanostructures is discovered and addressed in terms of the thickness-dependent and anisotropic surface diffusion of adatoms, as well as the strain relaxation, on the pillar. The comprehensive scenario of Ge growth on the Si nanopillar is proposed, which well accounts for the experimental results. It sheds light on the controllable growth of nanostructures on the pillars. Moreover, it inspires the prominent two-step growth procedure to feasibly engineer the desired pillar-QN semiconductor system. Our results open a promising door to the deliberately designed pillar-QN semiconductor system, QDM and QR, which will facilitate the fundamental studies on the cavity quantum electrodynamics and the fabrication of the innovative nano-optoelectronic devices.

## Methods

### Fabrication of scalable array of ordered Si nano-pillar

The ordered Si nano-pillars in a large area is fabricated via Nanosphere lithography. Firstly, A monolayer of PS spheres (Duke Scientific Corp.) arranged in a hexagonal lattice can be obtained on a Si (001) substrate using the modified Langmuir-Blodgett technique^24^, as typically shown in Fig. 1a. Such ordered PS spheres serve as the mask for the subsequent fluorine-based inductively coupled plasma reactive ion etching (ICP-RIE; Oxford PlasmaLAB ICP180) using a mixture gas of SF_6_ and C_4_F_8_. The flow rate of SF_6_ and C_4_F_8_ are 25 and 50 sccm, respectively. The RIE and ICP power is 30 W and 1200 W, respectively. The chamber pressure is 15 mTorr, and substrate temperature is 295 K. The ordered Si pillars with a reduced PS sphere on the top of each pillar are then obtained, as typically shown in Fig. 1b. By using Bosch process that consists of cyclic etching and passivation steps (SF_6_ for etching, C_4_F_8_ for passivation), the maximum achievable pillar height can be about 5 μm. After etching the remained PS spheres by RIE using oxygen as reactive gas, an array of ordered Si pillars can be obtained, as typically shown in Fig.1c.

### Growth of Ge on ordered Si nano-pillar

The pillar templates are subsequently employed for the controllable growth of self-assembled Ge nanostructures by a solid source molecular beam epitaxy (MBE; Riber Eva-32). They are chemically cleaned using RCA method and a subsequent HF treatment to obtain hydrogen-terminated surface before loading into the MBE chamber. After the *in-situ* thermal desorption at 800 °C for 3 min, a Si buffer layer of 50 nm was grown at a growth rate of 0.6 Å/s while ramping the temperature from 430 °C to 480 °C. A growth interruption is applied for *in-situ* rapid thermal annealing at 600 °C for 1 minute to obtain a smooth surface. Finally, different amount of Ge is deposited at the temperature in the range of 480 °C to 580 °C. Moreover, two types of growth procedures are adopted for the controllable growth of Ge. One is the general one-step procedure with a constant growth rate of about 0.025 Å/s at 520 ºC. We develop another two-step growth procedure. The growth conditions in both steps are deliberately adjusted to intentionally control the self-assembled QNs on the pillars.

### SEM, AFM and TEM measurement

The arrangement of PS spheres and the patterned pillars are observed by SEM (Zeiss Sigma). The surface morphologies of self-assembled Ge nanostructures on Si pillars are investigated ex-situ by AFM (Veeco DI Multimode V SPM) in a tapping mode. XTEM was performed on a FEI TECNAI G^2^ S-TWIN F20 operating at 200 kV.

## Additional Information

**How to cite this article**: Wang, S. *et al*. Evolution and Engineering of Precisely Controlled Ge Nanostructures on Scalable Array of Ordered Si Nano-pillars. *Sci. Rep.* **6**, 28872; doi: 10.1038/srep28872 (2016).

## Supplementary Material

Supplementary Information
