# Supplementary material for: Evolution and Engineering of Precisely Controlled Ge Nanostructures on Scalable Array of Ordered Si Nano-pillars
Source: Sci Rep. 2016 Jun 29;6:28872. doi: 10.1038/srep28872 (PMC4926084; doi:10.1038/srep28872)
Supplement: Supplementary Information [file srep28872-s1.pdf]

SUPPLEMENTARY INFORMATION FOR

Evolution and Engineering of Precisely Controlled Ge Nanostructures on Scalable Array of Ordered Si Nano-pillars

Shuguang Wang[1], Tong Zhou[2], Dehui Li[3], and Zhenyang Zhong[1,*]

[1]State Key Laboratory of Surface Physics and Department of Physics, Collaborative Innovation Center of Advanced Microstructures, Fudan University, Shanghai 200433, China

[2]School of Science, Shandong University of Technology, Zibo 255049, China

[3]Shanghai Institute of Applied Physics, Chinese Academy of Sciences, Shanghai 201800, China

[*]zhenyangz@fudan.edu.cn

Self-assembled Ge quantum dots (QDs) on the normal flat Si (001) substrates are also realized under the same growth conditions as those for the controllable Ge quantum nanostructures (QNs) on the Si nano-pillars. Figure S1a-c show the AFM images of the surface morphologies after 1.8, 2.0 and 2.2 nm Ge deposition on the flat Si (001) substrates, which is grown via one-step growth procedure at 520 ºC and a growth rate of 0.025 Å/s, respectively. It can be seen that both small pyramid-like and large dome-like Ge QDs are obtained. With the increase of the Ge deposition, the small pyramid-like QDs tend to evolve into large dome-like QDs, and more super-dome-like QDs appear. In addition, the density of QDs is slightly decreased with the increase of the Ge deposition due to the coarsening process. Figure S2 shows the AFM images of the surface morphologies after 1.8 nm Ge deposition on the flat Si (001) substrates via the two-step growth procedure. It can be seen that the self-assembled Ge QDs on the flat Si (001) substrates are considerably affected by the growth conditions. The characteristics of the self-assembled QDs in Figure S2a-c demonstrate that the Ge growth in the second step dominates the formation of QDs on the flat Si (001) substrates. All these self-assembled Ge QDs on the flat Si (001) substrates are spatially random.

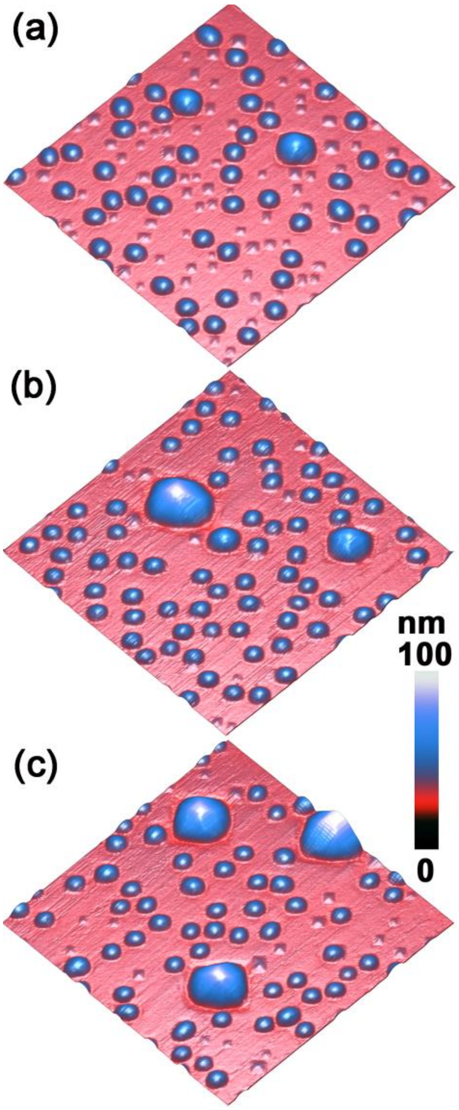

Figure S1: AFM images (1×1 μm^2) of self-assembled Ge QDs on the reference flat Si (001) substrates, corresponding to Figure 2, via the one-step procedure at 520 ºC and a Ge growth rate of 0.025 Å/s with nominal Ge deposition of, a) 1.8 nm, b) 2.0 nm, c) 2.2 nm. The color bar is shown at the right.

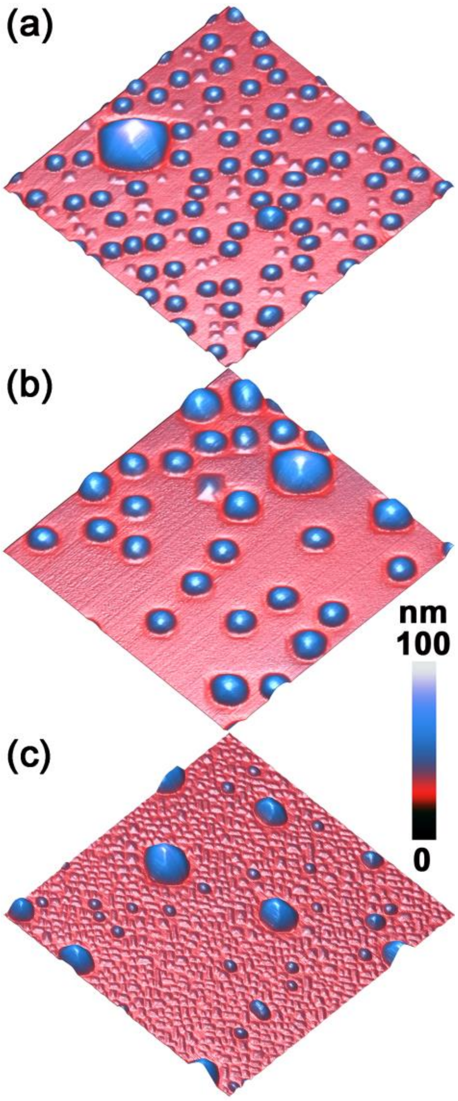

Figure S2: AFM images (1×1 μm^2) of self-assembled Ge QDs on the reference flat Si (001) substrates, corresponding to Figure 3, via the two-step procedure with the Ge deposition of, a) (0.8 nm at 500 ºC and 0.1Å/s) + (1.0 nm at 520 ºC and 0.025Å/s), b) (0.8 nm at 500 ºC and 0.1Å/s) + (1.0 nm at 580 ºC and 0.025Å/s), c) (0.8 nm at 580 ºC and 0.025Å/s) + (1.0 nm at 480 ºC and 0.1Å/s). The color bar is shown at the right.
